# Integration of Core Mechanisms Underlying Plant Aerial Architecture

**DOI:** 10.3389/fpls.2021.786338

**Published:** 2021-11-18

**Authors:** Marcus G. Heisler

**Affiliations:** School of Life and Environmental Science, University of Sydney, Camperdown, NSW, Australia

**Keywords:** plant, development, patterning, mechanics, auxin, polarity, differentiation, microtubules

## Abstract

Over the last decade or so important progress has been made in identifying and understanding a set of patterning mechanisms that have the potential to explain many aspects of plant morphology. These include the feedback loop between mechanical stresses and interphase microtubules, the regulation of plant cell polarity and the role of adaxial and abaxial cell type boundaries. What is perhaps most intriguing is how these mechanisms integrate in a combinatorial manner that provides a means to generate a large variety of commonly seen plant morphologies. Here, I review our current understanding of these mechanisms and discuss the links between them.

## Introduction

Vascular plants create some of the most striking and diverse architectures in biology. From the fractal-like architectures of fern leaves to the spiral patterns of sunflowers. At the same time, many morphological characteristics are largely conserved, including the flat shape of leaves, the cylindrical shape of stems, and the periodic patterns of organogenesis from the shoot apex. Are there a core set of developmental mechanisms that can potentially explain much of this morphology? In this review, I discuss our current understanding of several such mechanisms and, in particular, how they integrate with one another. These include feedback between mechanical stresses and cellulose orientations *via* interphase microtubule arrays, auxin and its transport, the role of adaxial-abaxial cell type boundaries, and, lastly, tissue differentiation. The broad coverage of these topics necessarily means that detail may be limited, so I apologize in advance for the many important references omitted. The aim is to clarify the big picture by focusing on a minimal set of key concepts and how they are linked to one another. In this way, I hope a deeper understanding of the broader patterns of plant morphology become apparent.

## A Mechanical Stress, Cellulose Fibril Feedback Loop Patterns Plant Morphogenesis

Plant cells are encased within a rigid cell wall made from cellulose, pectin, xyloglucans, and other carbohydrates. Like the skin of a balloon, the cell wall prevents plants cells from bursting due to turgor pressure. Unlike a balloon, plant cell walls also behave plastically to enable growth. Cell wall expansion is restricted by cellulose microfibrils which are strong, stiff, and cross-linked. Hence, the orientation of these microfibrils is critical in determining how easily cell walls can expand and grow in different directions. In turn, the orientation of cellulose microfibrils depends on the orientation of interphase microtubules, which guide the trajectories of cellulose synthase enzyme complexes within the plasma membrane (Gutierrez et al., 2009). Feedback from maximal tension directions to cellulose fibril orientations was proposed as early as the 1930s (Castle, 1937; Van-Iterson, 1937) and even taken for granted by the 1950s (Roelofsen, 1950). However, the hypothesis was largely rejected in the 1970s as it was assumed principle stress directions could not be sensed independently of cell wall strains (Gertel and Green, 1977). Indeed, how principle directions of stress can be sensed independently of wall strains associated with growth remains speculative (Williamson, 1990; Hamant et al., 2019). In any case, recent work has provided additional evidence strongly supporting a role for mechanical stresses in orienting microtubules (and by extension cellulose microfibrils) and that this regulation represents a core mechanism for enabling plant tissues to reinforce themselves along principle stress directions and shape their growth.

**Figure 1:**
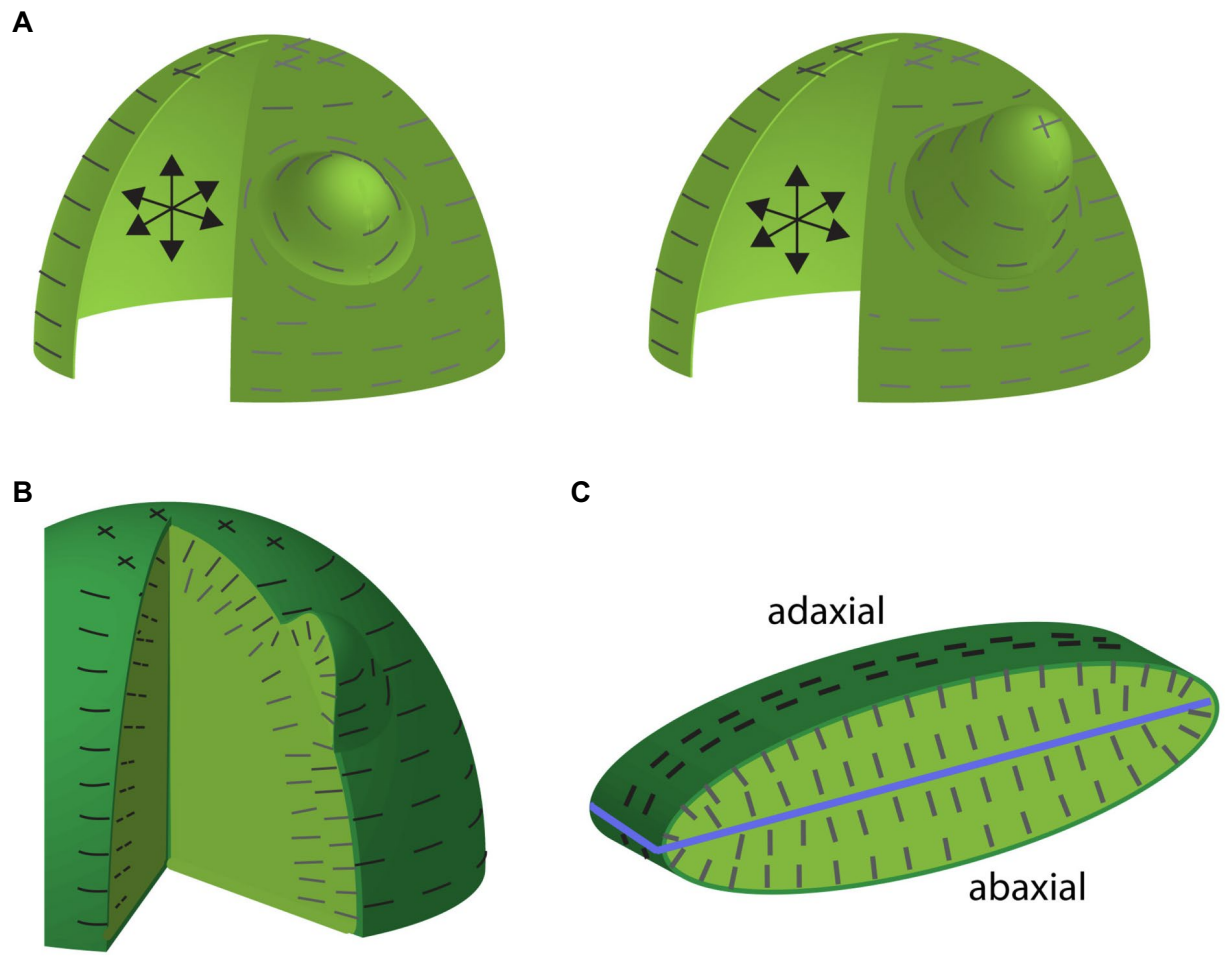
Maximal tension predictions for shell and multicellular-layered mechanical models of the shoot apical meristem (SAM) including organ emergence. **(A)** Depictions of a pressurized shell model of the SAM and an emerging lateral organ early (left) and later (right) during development. Internal pressure is depicted by the black arrows. Predicted maximum tension orientations along the surface are depicted by gray lines. Note that anisotropic circumferential orientations toward the SAM periphery compared to isotropic stresses toward the apex. Maximum tension orientations are circumferential around the primordium, where wall loosening has occurred. Note that these stress orientations match observed microtubule and cellulose orientations. Circumferential cellulose orientations around the organ primordium would be expected to promote proximodistal growth. **(B)** Depiction of maximal tension orientations for a multicellular-layered SAM and primordium. The degree to which tensions in this model match those of the shell model depends on the relative stiffness of the outer epidermal cell wall compared to inner cell walls. Maximal tensions along inner anticlinal walls are oriented anticlinally (perpendicular to the SAM surface) matching observations. **(C)** Depiction of maximal tension orientations (dark lines) in a cross section of multilayered mechanical model of a leaf. Light green marks internal tissues, blue line marks ad-ab boundary. Note that predicted internal tension orientations are aligned largely perpendicular to the ad-ab boundary (although not so much at the leaf margin) and match observed microtubule and cellulose orientations.

The evidence supporting the existence of a mechanical stress/microtubule/microfibril feedback loop (from here on abbreviated to stress/cell wall feedback loop) in the shoot apical meristem (SAM) primarily comes from observations that microtubules and cellulose microfibril orientations correlate with predicted maximal tension directions as well as mechanical perturbation experiments demonstrating reorientations in both microtubule and cellulose orientations that are in correspondence with the applied maximal tension (Hamant et al., 2008). In addition, cell division planes have been found to follow predicted stress orientations, thereby further reinforcing plant tissues against tensile stress due to the insertion of new wall material (Louveaux et al., 2016). Importantly, the stress patterns predicted by pressurized shell models, which match observed microtubule orientations, now have experimental support from the finding that when cell-cell contacts are weakened, the resulting cracks and cell separations occur oriented along directions predicted by mechanical models (Verger et al., 2018). What are the consequences of this stress/cell wall feedback loop for plant morphogenesis? There are several important implications. Firstly, supracellular tensile stress patterns provide a means for plant tissues to coordinate the orientation of both cell division planes and cellulose microfibril orientations over relatively large distances. Secondly, if cellulose fibrils and cell walls are oriented to resist tension, this allows the plant to reinforce itself structurally in response to anisotropic forces, i.e., organs or tissues are physically more robust (Bozorg et al., 2014). Lastly, orienting cellulose microfibrils along maximal tension directions means that there is minimal cellulose reinforcement in orthogonal directions, which may promote growth in orthogonal directions. This latter point in particular is expected to enable initial asymmetries in shape to be amplified, potentially explaining some of the common plant organ shapes we see. For example, if a pressurized shell model of the SAM is loosened locally, a bulge forms and maximal tensions orient circumferentially around the bulge (Hamant et al., 2008). This not only matches observed microtubule arrangements but also predicts the formation of a new growth axis orthogonal to the SAM surface (Figure 1A). Conceptualizing a newly formed primordium as a pressurized shell, circumferential stresses would be expected to stabilize cellulose microfibril orientations in a transverse arrangement and thereby promote a robust extension of the new growth axis proximodistally (Bozorg et al., 2014; Figure 1A).

Apart from finding that the stress/cell wall feedback loop operates in additional tissue contexts (Sampathkumar et al., 2014; Hervieux et al., 2017; Belteton et al., 2021), an important recent advance has been in developing mechanical models to simulate more complex tissue structures. Rather than assuming pressurized shells, multilayered tissue models have been constructed and compared to simpler models (Ali et al., 2019). Perhaps the most important conclusion from this work has been that the degree to which multilayered models generate similar surface stress patterns to those of shell models depends on the relative stiffness of inner cell layer walls compared to the outer epidermal cell layer walls. Considering known estimates of the thickness of the *Arabidopsis* SAM outer cell wall, the authors estimated a stiffness ratio of outer to inner walls to be in the range of 3–10:1. Given these ratios, simulations demonstrate that the periclinal stress patterns (i.e., parallel to the tissue surface) in the outer walls of multilayered SAM models resemble those produced by shell models, thus validating earlier approaches (Ali et al., 2019). A second important conclusion from this work is that maximal stress patterns in the anticlinal walls (i.e., perpendicular to the tissue surface) of both the epidermis and underlying cell layers are predominantly oriented anticlinally (Ali et al., 2019), again matching observed microtubule and cellulose orientations (Sakaguchi et al., 1988; Figure 1B). This *a priori* indicates that loosening the walls of subepidermal cells or randomizing cellulose orientations may result in anticlinal cellular growth, which would account for the initial outward bulge associated with lateral organ formation (see further discussion on auxin’s influence on microtubules see below).

A multilayered cellular mechanical model similar to that used for the SAM above has been applied to understanding stress patterns in developing leaves (Zhao et al., 2020). In a elongate and flattened ellipsoid-shaped leaf model consisting of six cell layers pressurized by turgor, the principle stresses at the surface were found to be circumferential (with respect to the proximodistal axis), similar to models of a stem. Internally, the maximal tensions on anticlinal walls were largely oriented across the adaxial-abaxial axis due to the flattened shape (Zhao et al., 2020; Figure 1C). These orientations also match observed microtubule and cellulose orientations in very young leaf primordia, although the orientations may be better described as anticlinal (rather than aligning along the adaxial-abaxial axis), judging from cellulose and microtubule orientations at leaf margins (Green and Lang, 1981; Sakaguchi et al., 1988; Sylvester et al., 1989; Zhao et al., 2020). However, at slightly older stages, observed microtubules were found not to match model predictions. For the model, stress patterns remained circumferential at the surface and oriented along the proximo-distal axis at the interface between the L1 and L2 cell layers. In contrast, observed microtubules became isotropic in these regions (Zhao et al., 2020). To explore the significance of these different microtubule scenarios for leaf shape, the authors compared how the model behaved without stress/cell wall feedback, with stress/cell wall feedback applied to all cell walls and with stress/cell wall feedback applied to all cell walls except the outer wall of the epidermis. Without stress/cell wall feedback at all, the structure thickened, transforming toward a spherical shape. When stress/cell wall feedback was implemented throughout, the model expanded and flattened more compared to its original slightly flattened shape. Implementing the stress feedback mechanism in all but the outer cell walls resulted in an even more flattened shape compared to the starting point and also made stress isotropic at the interface between the L1 and L2 cell layers, matching the observed microtubules. Thus, the authors conclude that leaf flatness is promoted by the stress feedback mechanisms and a specific decoupling of the stress feedback mechanism in the tangential walls of the leaf epidermis (Zhao et al., 2020). Importantly, however, in order for the stress/cell wall feedback loop to promote flattening, the model template had to be somewhat flattened to begin with. This leaves open the important question of how leaf primordia are initially flattened, as will be discussed further below.

To summarize, considerable evidence supports the existence of a mechanism that enables plant cells to sense principle stress directions in their walls and utilize this information to orient cellulose fibril orientations *via* the regulation of cortical interphase microtubule arrays. Modeling and experiments demonstrate that this feedback system maintains and reinforces plant structures against anisotropic stresses and, in doing so, orients and stabilizes growth directions. Modulation of the system in particular cell walls or at specific locations is predicted to further differentially regulate growth and generate new growth axes, respectively.

## Auxin, the Cell Wall Disruptor

Given that a mechanical stress feedback-system operates to physically stabilize plant structures, how are changes to growth patterns brought about in order to initiate and position new grow axes, e.g., flowers and leaves? The plant hormone auxin plays a central role in this process. Over 80years ago, it was found that auxin applied exogenously to Lupin apices induced leaf growth (Snow and Snow, 1937). More recently, it was demonstrated that *Arabidopsis* plants disrupted for auxin transport failed to form flowers (Okada et al., 1991) and that local auxin application to the meristem of these plants could rescue this defect (Reinhardt et al., 2000). These observations imply that the distribution of auxin at the shoot apex determines where organs form and that auxin transport is required for this. By assessing the polar localization patterns of the auxin efflux carrier PIN-FORMED1 (PIN1), it was subsequently demonstrated that PIN1 likely transports auxin directly to sites of organ inception (Reinhardt et al., 2003). Auxin is well-known to alter cell wall properties. For instance, in some tissues, auxin triggers cellular proton efflux and a consequent lowering of apoplastic pH. Low pH is turn alters the activity of cell wall modifying enzymes such as expansins (Mcqueen-Mason et al., 1992) and pectin methyl esterases (PMEs; Hocq et al., 2017). Acidification of the apoplast is mediated by short-lived proteins encoded by a large family of SMALL AUX UP-RNA (SAUR) genes. Auxin induces the expression of SAUR proteins, which activate the PM H+−ATPase by binding to and inhibiting the activity of PP2C phosphatases (Du et al., 2020). While a role for SAUR proteins in organ initiation has not yet been demonstrated, local application of either expansin or PME to plant meristems is sufficient to trigger organ growth, while constitutive expression of a PME inhibitor blocks organ growth (Fleming et al., 1997; Peaucelle et al., 2008). Measurements using atomic force microscopy also indicate that the walls of subepidermal cells become more elastic at sites of organ formation as well as after treatment with either PME or auxin (Peaucelle et al., 2011; Braybrook and Peaucelle, 2013). All together, these findings indicate that auxin likely triggers organ growth in part by modifying cell wall properties.

Another way in which auxin promotes organ growth is by disrupting the stability of interphase microtubule arrays, which in turn is expected to disrupt cellulose orientations. As discussed above, interphase microtubule arrangements form supracellular patterns aligned along maximal cell wall tensions. However, close examination of these patterns reveals that at sites of organ inception, where auxin accumulates, microtubule orientations are disrupted. Furthermore, auxin application experiments demonstrate that high auxin levels are sufficient to cause such microtubule disruptions (Sassi et al., 2014). By applying the microtubule depolymerizing drug oryzalin to *pin1* apices, it was also found that microtubule disruption alone, without auxin, could induce organ formation (Sassi et al., 2014) implying that organ formation is promoted by a shift from anisotropic to isotropic cellular mechanical properties (Sassi et al., 2014). Building on this work, a follow up study identified a set of genes encoding cell wall remodeling enzymes that are expressed in organ primordia that form in response to microtubule disruption (Armezzani et al., 2018). Surprisingly, by monitoring the expression of the auxin transcriptional marker DR5, it was shown that when organs initiate in response to microtubule disruption, auxin signaling is not triggered, implying that auxin signaling is also not involved in promoting the expression of the associated cell wall enzymes. Lastly, this study also demonstrated that the application of PME to the SAM, which previously had been shown to induce organ outgrowth (Peaucelle et al., 2011), was also sufficient to induce microtubule disruption (Armezzani et al., 2018). All together these findings demonstrate that auxin promotes the formation of new growth axes, i.e., leaves and flowers, in part by triggering both microtubule disorganization and the expression of cell wall remodeling enzymes and that these processes reinforce each other.

## Transcription Factors Involved in Auxin Triggered Organogenesis

What factors act to promote organ formation downstream of auxin? In *Arabidopsis*, a critical transcription factor that acts downstream of auxin is Auxin Response Factor 5 (ARF5) also named as MONOPTEROS (MP; Przemeck et al., 1996; Guilfoyle et al., 1998; Hardtke and Berleth, 1998). MP regulates the transcription of target genes by recruiting SWI/SNF chromatin remodeling ATPases to their promoters in the presence of auxin (Wu et al., 2015). *mp* mutants form a flowerless inflorescence apex demonstrating that *MP* is necessary for flower formation (Przemeck et al., 1996). Combining mutations in *MP* with mutations in the *NPH4* gene, which encodes a related Auxin Response Factor ARF7, results in a more severe phenotype, similar to that of *mp* mutants treated with the auxin transport inhibitor NPA (Hardtke et al., 2004; Schuetz et al., 2008; Carey and Krogan, 2017). Such plants fail to form both flowers and leaves. Similarly, mutations in *MP*, *ARF3*, and *ARF4* also fail to produce flowers and leaves (Chung et al., 2019), indicating a broad requirement for ARF function and therefore auxin-induced transcription, for plant organogenesis.

So far, several MP target genes that promote flower formation have been identified in *Arabidopsis* including *AINTEGUMENTA* (*ANT*), *AINTEGUMENTA-LIKE6* (*AIL6*). *PLETHORA* (*PLT*), *FILAMENTOUS FLOWER* (*FIL*), and *LEAFY* (*LFY*). Although mutations in these genes individually do not cause a dramatic loss of organs, several studies show they work together in a redundant fashion to promote flower formation downstream of auxin and *MP* (Li et al., 2013; Yamaguchi et al., 2013, 2014, 2016; Wu et al., 2015). LFY functions in part by promoting auxin signaling, although it also reduces auxin synthesis (Li et al., 2013). LFY also directly promotes *ARF3* or *ETTIN* (*ETT*) expression (Yamaguchi et al., 2014), which as discussed above, acts with MP and ARF4 to promote organ development. In part, ETT, ARF4, and MP promote flower formation by repressing *STM* and *BP* (Chung et al., 2019) expression. *STM* and *BP* are normally downregulated at organ initiation sites and while this downregulation is not essential for organogenesis, their extopic expression suppresses organ differentiation (Lenhard et al., 2002; Aguilar-Martinez et al., 2015) and enhances the organ-less phenotype of weak *mp* alleles (Chung et al., 2019). MP regulates *STM* and *BP* expression through the intermediary FIL, while ETT and ARF4 repress *STM* expression directly (Kumaran et al., 2002; Chung et al., 2019).

Another set of auxin-induced genes encoding transcription factors critical to organ formation are *LEAFLESS* (*LFS*) in tomato and its two orthologs *DORNRONSCHEN* (*DRN*) and *DRN-like* in *Arabidopsis* (Capua and Eshed, 2017). Both *lfs* and *drn drnl* double mutants fail to form any lateral organs including cotyledons, demonstrating these genes play a central role in organ formation (Capua and Eshed, 2017). Exactly, how these genes promote organ formation is still unclear although there is a link to cytokinin since ectopic *DRN* expression promotes cytokinin-independent shoot regeneration in culture and several cytokinin signaling genes are mis-regulated in the mutant (Banno et al., 2001; Capua and Eshed, 2017).

Perhaps the most enigmatic set of auxin-induced genes involved in lateral organ formation are the *WUSCHEL-RELATED HOMEOBOX* (*WOX*) genes. *PRESSED FLOWER* (*PRS*), in particular, is expressed in the peripheral zone of the vegetative shoot and at flower initiation sites, possibly together with *WOX4* (Caggiano et al., 2017; Eeda and Werr, 2020). When auxin transport is partially compromised, *prs* mutants fail to form leaves and resemble *mp nph4* mutants (Nakata et al., 2018). *PRS* and *WOX4* are also expressed in developing leaves together with *WOX1* and *WOX5* (Nakata et al., 2012; Eeda and Werr, 2020; Zhang et al., 2020). *WOX1*, *PRS*, and *WOX5* have been shown to work together to promote leaf lamina expansion (Zhang et al., 2020). Relatedly *WOX4* expression in the vasculature is required to promote cambial growth in response to auxin. The role of WOX transcription factors will be discussed further below.

## Plant Adaxial-Abaxial Boundaries Act Globally to Localize Auxin-Dependent Growth

**Figure 2:**
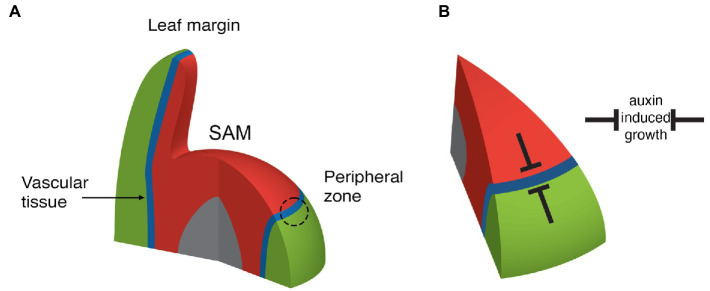
Depiction of adaxial (red), abaxial (green), boundary (blue), and pith (grey) regions near the SAM. **(A)** Note that organ founder cells are centered on the ad-ab boundary within the peripheral zone (dashed circle). The boundary extends from the SAM epidermis internally, where it is associated with the formation of the stem vascular tissue. The boundary also extends into new leaves to their margin. **(B)** Adaxial and abaxially expressed transcription factors repress auxin induced growth such that organs can only form on the boundary.

Somehow plants must confine auxin-induced growth activity to well-defined regions in order to regulate their architecture. Plant aerial tissues that respond to auxin in terms of localized growth include the SAM peripheral zone (Reinhardt et al., 2000), where new leaves or flowers arise, the margins of leaves, where serrations or leaflets can develop (Scarpella et al., 2006; Koenig et al., 2009) and the vascular cambium, which is responsible for vascular cell proliferation and stem thickening (Suer et al., 2011). All three of these regions can be related to one another in that they are contiguous and centered in between the expression domains of genes encoding adaxial class III homeodomain-leucine zipper (HD-ZIPIII) and abaxial KANADI (KAN) transcription factors, from here on termed adaxial-abaxial (ad-ab) boundaries (Emery et al., 2003; Caggiano et al., 2017; Figure 2A). New organ primordia are centered on the SAM ad-ab boundary at initiation and as these new organs grow, the ad-ab boundary is maintained and propagated within them (Figure 2A). The SAM ad-ab boundary in turn represents the epidermal extension of an ad-ab boundary that runs internally within the stem, corresponding to where xylem, cambium, and phloem develop. The overall pattern of adaxial and abaxial expression is established during embryogenesis (Kerstetter et al., 2001; Mcconnell et al., 2001). What is the significance of this arrangement? In the SAM, if the *HD-ZIPIII* or *KAN* genes are ectopically expressed throughout the peripheral zone, organ formation is suppressed (Caggiano et al., 2017). Conversely, in multiple mutants for the *HD-ZIPIII* and *KAN* genes, outgrowths develop ectopically from the meristem center or abaxial surface of the hypocotyl, respectively, (Izhaki and Bowman, 2007; Zhang et al., 2017). Similarly, for leaves, ectopic expression of either *KAN* or *HD-ZIPIII* genes represses lamina growth while loss of both *KAN1* and *KAN2* function or loss of the adaxial transcription factor AS2, can lead to ectopic leaf-like outgrowths from the abaxial (Eshed et al., 2004) and adaxial leaf surfaces, respectively (Alvarez et al., 2016). The cambium also lies between the expression domains of HD-ZIPIII genes in the xylem and *KAN* genes in the phloem (Ilegems et al., 2010). Loss of *HD-ZIPIII* function leads to a switch from xylem quiescence to cell proliferation and expansion of auxin signaling (Ilegems et al., 2010; Smetana et al., 2019) while ectopic expression of *HD-ZIPIII* genes in the cambium leads to a cell-autonomous quiescence (Smetana et al., 2019). Like-wise, the loss of *KAN* function leads to increased cell proliferation, this time in the cambium and pericycle while ectopic KAN1 suppresses cell proliferation by repressing PIN1-mediated auxin transport (Ilegems et al., 2010). In all three regions, therefore, HD-ZIPIII and KAN transcription factors repress cell proliferation and growth, where they are expressed (Figure 2B). Accordingly, genes that promote growth are expressed most strongly in between the *HD-ZIPIII* and *KAN* expression domains, i.e., at the ad-ab boundary, in all three different contexts. For instance *ANT* expression marks a contiguous domain from sites of organ emergence to the vascular cambium and middle domain of the leaf. (Elliott et al., 1996; Long and Barton, 2000; Smetana et al., 2019). It is important to note, however, that while a maximal growth response is centered in between the *HD-ZIPIII* and *KAN* expression domains (i.e., at the boundary), it is not an all or nothing response. During leaf formation, for instance, organ founder cells include cells adjacent to the boundary, i.e., in the *HD-ZIPIII* and *KAN* expression domains and this enables the propagation of the ad-ab boundary into the growing organ (Caggiano et al., 2017). Perhaps similarly, the cambium xylem and phloem projenitor cells, adjacent to the stem cells, proliferate to form the phloem and xylem (Shi et al., 2019).

Despite the above mentioned similarities, many differences exist between ad-ab boundaries within the different tissue contexts mentioned. For instance, the *YABBY* genes are required exclusively in leaves to maintain proper boundary function and prevent the expression of meristem associated genes (Sarojam et al., 2010). However, such differences may not be as extensive as first assumed. For instance, while *WOX4* has been associated primarily with vascular tissues, a recent study indicates that it may also function in the PZ and leaf (Eeda and Werr, 2020). A comparative approach therefore holds great promise not only for understanding these boundaries within each context but also for understanding how the system as a whole has evolved.

## How do Ad-Ab Boundaries Localize Auxin Responsive Growth in the Shoot and Leaf?

In the *Arabidopsis* SAM, auxin applied broadly to the surface only elicits a growth response at the peripheral zone, i.e., the SAM ad-ab boundary (Reinhardt et al., 2000). This suggests that auxin itself need not be restricted to the boundary for the growth response to be localized and implies that either general auxin signaling components are localized at the boundary or additional contingent factors are. Consistent with the former possibility, auxin signaling in the SAM and leaves, as indicated by the DR5 reporter, is somewhat restricted compared to the broader auxin distribution detected by the fluorescent auxin signaling sensor DII (Vernoux et al., 2011; Bhatia et al., 2016; Caggiano et al., 2017; Guan et al., 2017; Galvan-Ampudia et al., 2020). Consistent with this, auxin signaling components, such as IAA proteins and ARFs, including MP/ARF5, are more highly expressed in the PZ compared to the neighboring CZ due to their repression by the stem cell promoting factor WUSCHEL (WUS; Vernoux et al., 2011; Ma et al., 2019). Ectopic expression of MP or more potently, a constitutively active version of MP within the SAM central zone (CZ) causes the CZ to become transcriptionally responsive to auxin, as judged by DR5 expression (Ma et al., 2019). However, even under these circumstances, no ectopic organs form (Ma et al., 2019) indicating that the repression of organogenesis by adaxial and abaxial transcription factors extends beyond the regulation of general auxin signaling components.

As mentioned above, one set of factors clearly critical for auxin-induced cell proliferation are the *WOX* genes. In the leaf, *WOX1* and *PRS* expression is restricted to the leaf ad-ab boundary region, or middle domain where *HD-ZIPIII* and *KAN* expression is low or absent (Nakata et al., 2012; Caggiano et al., 2017). Although exogenous auxin application increases their expression levels *via* MP (Caggiano et al., 2017; Guan et al., 2017), their expression domains do not change (Caggiano et al., 2017), indicating adaxial and abaxial factors must restrict their expression. Abaxially, this is accomplished by *ARF2*, *ARF3*, and *ARF4* (Pekker et al., 2005; Alvarez et al., 2006; Guan et al., 2017) in conjunction with *KAN1* and *KAN2* (Nakata et al., 2012). These genes are normally expressed abaxially and in plants reduced for their function, *WOX1* and *PRS* are derepressed, resulting in outgrowths developing from abaxial tissues in a *WOX1*/*PRS* dependent manner (Nakata et al., 2012; Guan et al., 2017). Such outgrowths also form if *WOX1* is expressed abaxially under the *FIL* promoter in a wild-type background, demonstrating the potent capability of these genes to promote new growth axes (Nakata et al., 2012). Why *WOX* gene expression is absent on the adaxial side of the leaf is less clear. One proposal is that auxin levels are low in adaxial tissues, hence preventing *WOX* expression there (Guan et al., 2017). Evidence supporting this proposal comes in part from observations of the auxin sensor DII, which indicate lower levels of auxin in adaxial cells (Qi et al., 2014) and that constitutively active MP is sufficient to promote *WOX1* and *PRS* expression (Guan et al., 2017) adaxially. However, a difference in auxin levels between abaxial and adaxial tissues has been disputed (Bhatia et al., 2019) and, as mentioned above, auxin application experiments do not result in ectopic adaxial *WOX1* or *PRS* expression (Caggiano et al., 2017), arguing against low auxin levels being responsible for limiting their expression. Rather, there is evidence that *PRS* (at least) is repressed actively in adaxial tissues by the transcription factor AS2 (Alvarez et al., 2016). Similar to the situation in leaves, in the shoot *WOX4* and *PRS* are expressed roughly, where *REV* and *KAN1* expression is low (Caggiano et al., 2017; Yu et al., 2017; Eeda and Werr, 2020) and *PRS* function has been shown to be required for leaf formation in conjunction with auxin transport (Nakata et al., 2018). In the shoot, REV and KAN1 repress *PRS* expression indirectly and directly, respectively, (Ram et al., 2020).

How do the *WOX* genes promote growth? In part, this may be through maintaining the integrity of the adaxial-abaxial boundary in terms of gene expression (Nakata et al., 2012; Zhang et al., 2017). For instance, in the leaf margin (and perhaps elsewhere), *WOX1* and *PRS* are required to repress *miR165/166*, *FIL* and *AS2* expression. However, in addition, down-stream target analysis of the leaf-associated WOX1 protein has found that like WUS (Ma et al., 2019), many targets fall into the gene ontology term “response to auxin.” To further investigate, the influence of ectopically induced WOX1 alone, in combination with exogenous auxin or auxin alone were compared and it was found that WOX1 and auxin worked in an additive fashion to regulate common targets. This study also found that WOX1 induction together with simultaneous auxin application had a striking effect on cell proliferation. While auxin alone applied to the *Arabidopsis* hypocotyl caused lateral root-like structures to form with regular cell files, ectopic WOX1 in combination with auxin caused disorganized growth, with cell division planes appearing randomized. Thus, the authors propose different ratios of auxin to WOX gene activity may specify tissue specific growth patterns (Sassi et al., 2014).

Given the general role of *WOX* genes, such as *WUS*, *WOX5*, and *WOX4*, in maintaining quiescent stem cell niches, a role for *WOX1* and *PRS* in promoting growth appears somewhat contradictory. However, it may be consistent with the hypothesis that these genes act non-cell autonomously. For instance, both *WOX4* and *WUS* function in the cambium and SAM stem cell niche, respectively, to promote the division of adjacent stem cells nearby. Such a scenario is further supported by the fact that *WOX1* and *PRS* expression, driven by the *WUS* promoter, can complement the *wus* mutant in terms of meristem function, although shoot formation is delayed (Dolzblasz et al., 2016). In the context of leaf initiation and growth, this scenario would imply that leaf initials and marginal cells correspond to the stem cell niches of leaf marginal meristems, which is a concept that has recently received renewed support (Alvarez et al., 2016), as discussed further below. If *WOX* genes cell autonomously promote quiescence, how might they promote growth non-cell autonomously? Interestingly, *WOX1*, *PRS*, and *WOX5* have recently been shown to promote auxin synthesis *via YUC1* and *YUC4* expression in the proximal margin of the leaf. Furthermore, expressing *YUC1* using the *PRS* promoter largely rescues the *wox1 prs wox5* narrow leaf phenotype, although not the reduced overall size. In contrast, expressing *YUC1* using the *ML1* promoter throughout the epidermis did not rescue the narrow leaf phenotype, highlighting the role of the leaf margin in driving lateral organ growth (Zhang et al., 2020). Apart from promoting auxin biosynthesis, there is also evidence that WOX proteins are mobile (Yadav et al., 2011; Pi et al., 2015) and that their function depends on the interacting HAM proteins (Han et al., 2020). Hence, another possibility is that WOX proteins may activate or repress auxin-dependent growth in different cellular contexts, depending on HAM co-expression.

Although we have mainly focused on WOX transcription factors as promoters of auxin-induced growth that are restricted in expression to the ad-ab boundary, it is worth noting that in vascular tissues, transcription factors, such as TMO5/LHW and TMO5-likes (Miyashima et al., 2019), and the PEAR transcription factors (Miyashima et al., 2019) act downstream of auxin to promote periclinal cell division and growth. It will be interesting to investigate whether these genes play a similar role in lateral organ development at the SAM.

## A Periodic Pattern Generator Built Using a Coupling Between Mechanical Stress and Auxin Transport

**Figure 3:**
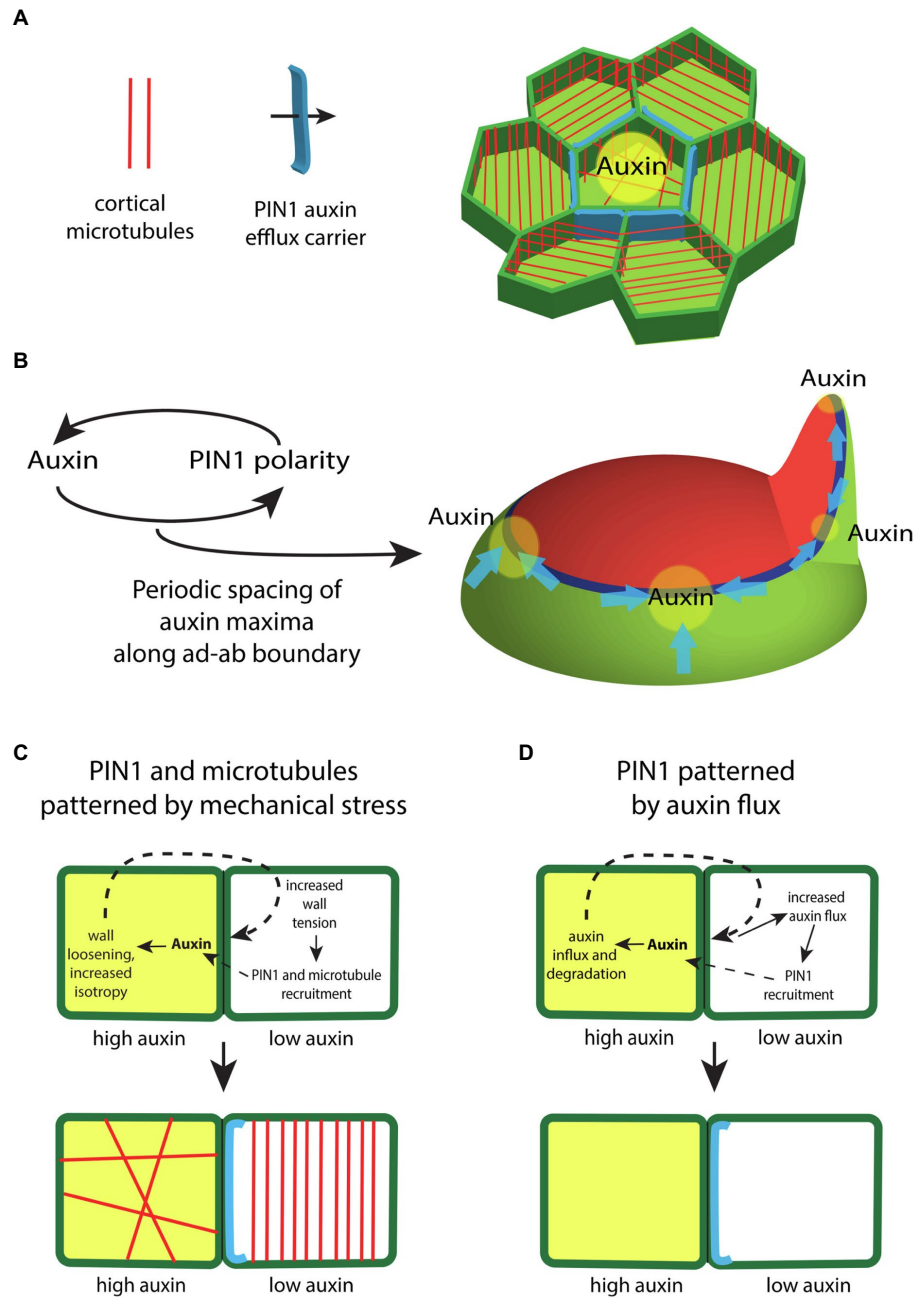
The regulation of auxin transport within the shoot epidermis. **(A)** In the shoot epidermis, the auxin efflux carrier PIN-FORMED1 (PIN1; blue) forms supracellular polarity convergence patterns that concentrate auxin locally. PIN1 localization within cells correlates with the orientation of interphase microtubule arrays (red), suggesting a role for mechanical stresses in regulating PIN1. **(B)** Auxin influences the polarity of PIN1 in a feedback loop. Modeling indicates this feedback is sufficient to generate a periodic spacing between PIN1 polarity convergence patterns (blue) and thus auxin maxima (yellow) along the ad-ab boundary (dark blue) located between adaxial (red) and abaxial (green) tissues. **(C)** Illustration depicting how differential auxin concentrations might regulate PIN1 and microtubule localization *via* changes to mechanical stress. High levels of auxin (yellow) in one cell triggers cell wall loosening. This leads to higher tension levels within the cell wall of an adjacent cell, which acts to polarize PIN1 (blue) and orient cortical microtubules (red). **(D)** Illustration depicting how high levels of auxin might regulate PIN1 *via* changes in auxin flux. High levels of auxin (yellow) triggers the expression of the auxin influx carrier as well as auxin degradation. This promotes an increase in diffusive auxin into the cell from adjacent cells. Increased auxin efflux in the adjacent cells promotes a corresponding PIN1 polarity (blue).

Given auxin responsive domains are established at boundary regions between adaxial and abaxial gene expression domains, how is auxin distributed within these regions to promote regular phyllotaxis? Auxin is transported directly to sites of organ emergence by the auxin efflux carrier PIN1, which forms supracellular polarity convergence patterns along the ad-ab boundary (Jonsson et al., 2006; Smith et al., 2006; Stoma et al., 2008; Cieslak et al., 2015; Abley et al., 2016; Hartmann et al., 2019). Interestingly, the mechanical stress feedback system that governs microtubule orientations appears to play an integral role in this process since mechano-sensitive interphase microtubule arrangements strongly correlate with PIN1 polarity patterns, while neither factor is dependent on the other for proper localization (Heisler et al., 2010; Figure 3A). This necessarily means a tight coupling between growth directions and PIN1 polarity and, in the context of polarity convergence points, serves to promote proximodistal growth oriented toward auxin maxima. Such patterns of growth are particular clear with respect to early leaf development (Kierzkowski et al., 2019).

Given the considerable evidence supporting a role for mechanical stress in orienting microtubules, the close alignment of PIN1 localization with microtubules implies mechanical stress helps to orient PIN1 polarity. Notably, other proteins, including BASL and BRXL2, also share a polarity axis with PIN1 and respond similarly to mechanical perturbations demonstrating that mechanical stress likely provides polarity information generally (Bringmann and Bergmann, 2017; Mansfield et al., 2018). If mechanical stress plays a role in orienting PIN1, auxin might also be expected to orient PIN1 since high concentrations of auxin likely alter stress patterns by disrupting interphase microtubule arrays and alterning the activity cell wall modifying enzymes, as mentioned above (Reinhardt et al., 2000; Zhao et al., 2020). Computational modeling has also demonstrated that with the right rules, feedback between auxin and its transport can generate the periodic spacing patterns typical of plant phyllotaxis (Jonsson et al., 2006; Smith et al., 2006; Stoma et al., 2008; Hartmann et al., 2019; Figure 3B). Experimental evidence supporting the type of feedback envisaged by these models comes from observing PIN1 polarity responses to local auxin application (Bayer et al., 2009) or clonal expression of *MP* (Bhatia et al., 2016). In these experiments, PIN1 polarity reorients in a convergence pattern toward applied auxin or toward *MP* expressing cells, thereby acting to increase local auxin signaling further in a positive feedback loop (Bhatia et al., 2016). Microtubules also orient circumferentially, maintaining their alignment with PIN1, suggesting auxin reorients the cell polarity axis by altering mechanical stresses (Bhatia et al., 2016). A similar response to auxin likely occur in the cambium where cell divisions are “organized” by *MP* expressing clones non-cell autonomously in a circumferential pattern (Smetana et al., 2019).

The question of exactly how auxin or MP influences PIN1 polarity is still not settled (Ten Tusscher, 2021). Apart from a lack of molecular understanding, modeling indicates that at least two alternative types of feedback from auxin can account for the observed response of PIN1 to local MP expression. If auxin loosens cell walls for instance, high levels of tension caused by this loosening can act as a polarity cue for neighboring cells (Heisler et al., 2010; Figure 3C). Alternatively, auxin acting through MP may promote auxin influx and degradation, thereby increasing auxin flux from neighboring cells, which might act as the polarity cue (Abley et al., 2016; Figure 3D). However, a recent study indicates that PIN1 polarity is relative insensitive to the polarity of neighboring cells arguing against auxin flux as a polarity determinant (Kareem et al., 2021).

## The Enigma of Leaf Morphogenesis

Previously, in relation to mechanical stress and microtubule orientations, we discussed leaf flattening and how it could be accounted for by mechanical stress patterns regulating microtubule and cellulose orientations across the leaf ad-ab axis (Figure 1C). However, it was found that such feedback could only promote flattening of the leaf if the leaf primordium was already somewhat flattened. How could a leaf primordium become flattened initially? One proposal in the literature is based on differences in measured stiffness of adaxial vs. abaxial tissues within the leaf primordium. It was found that adaxial tissues were stiffer than abaxial while a third intermediate domain was identified at later developmental stages (Qi et al., 2017). Computer simulations of a 2D mechanical model of a leaf primordium incorporating these mechanical differences appeared to demonstrate a flattening during growth as a result of differences in growth rates (Qi et al., 2017). However, it has been subsequently pointed out that the growth simulation in this study was not based on the commonly accepted principle that growth depends on cell walls yielding to a common turgor pressure. Instead, stiff walled cells were allowed to exert larger forces on their neighbors and therefore invade the domain of less stiff walled cells (Coen and Kennaway, 2018; Feng et al., 2018). Taking the same assumptions of stiffness but implementing a uniform turgor pressure assumption led to a very different but more intuitive result, calling into question the differential stiffness hypothesis (Coen and Kennaway, 2018). Is there another mechanism that might promote leaf flattening that could work in combination with mechanical feedback?

**Figure 4:**
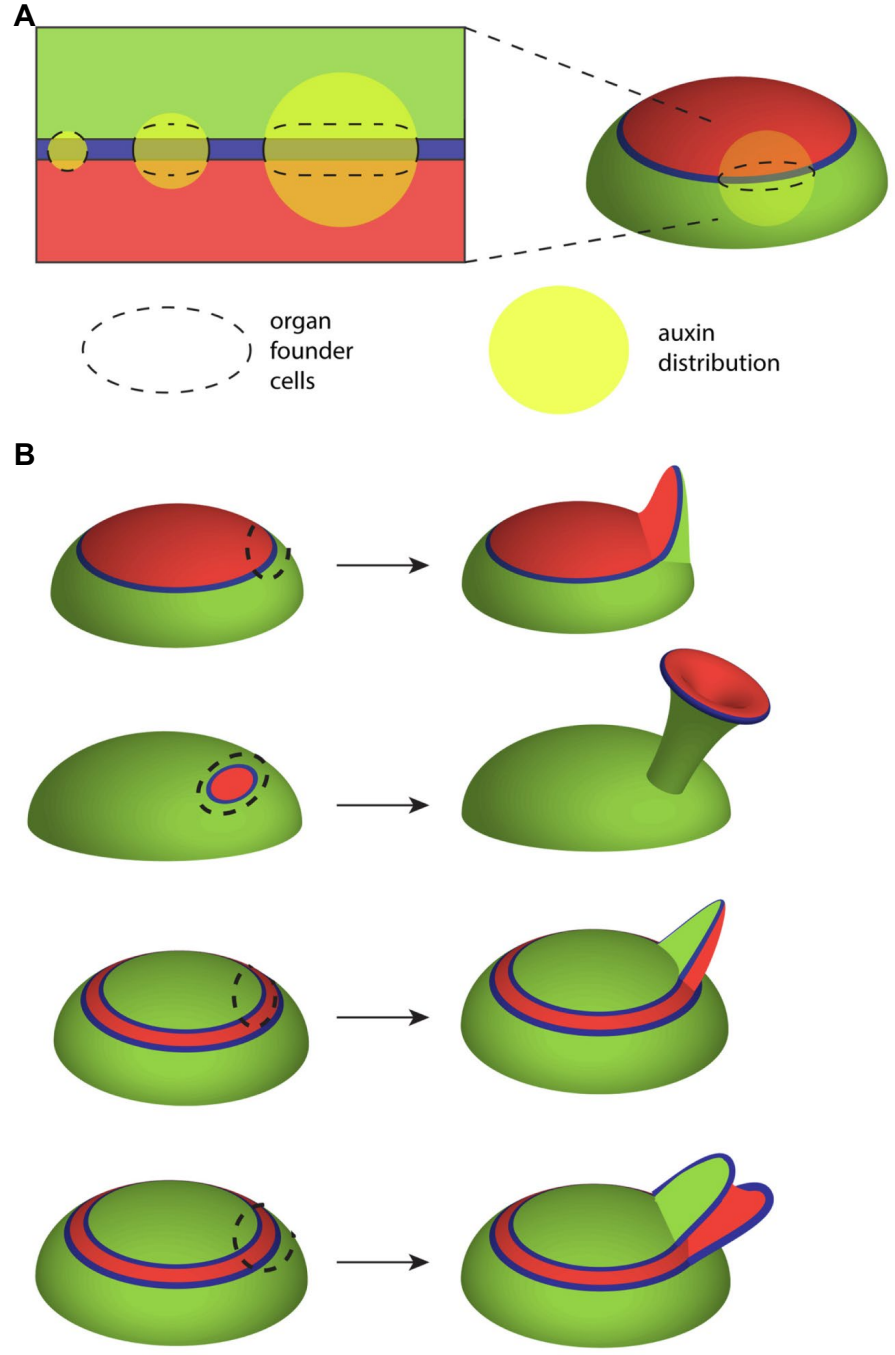
Influence of the ad-ab boundary on leaf growth. **(A)** Illustration showing how the shape of the auxin-responsive ad-ab boundary (blue) together with auxin (yellow) influences the configuration of organ founder cells (domain outlined by dashed line). As the boundary represents where response to auxin is maximized, cells farther away from the boundary are not recruited. As a consequence, while a broader auxin distribution promotes widening of the leaf primordium along the ad-ab boundary, its thickness across the ad-ab boundary remains unchanged. **(B)** Changes to the configuration of adaxial (red) and abaxial (green) cell types in the SAM mean that the spatial configuration of the auxin responsive boundary (blue) in relation to organ founder cells (dashed outline) changes. In turn, this can result in dramatic alterations to leaf morphology and cell type patterning. The top configuration shows wild-type development while the other configurations show alterations caused by ectopic expression of KAN1 in the SAM central zone.

**Figure 5:**
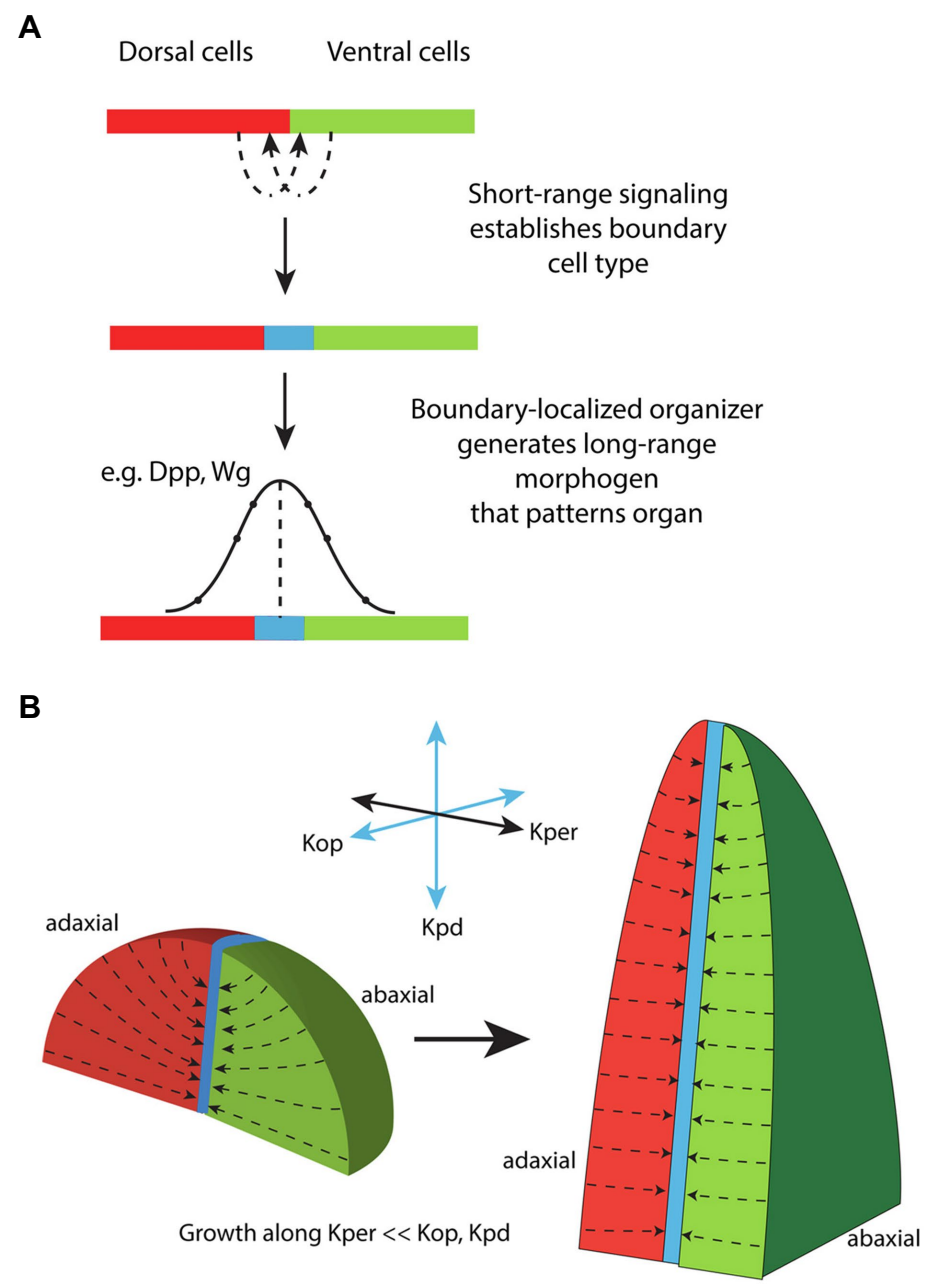
Proposals for how the ad-ab boundary might influence leaf morphogenesis. **(A)** Model for the establishment and function of a boundary-localized organizer in animals. Short-range signaling between dorsal (red) and ventral (green) cells results in the formation of a specialized boundary cell type (blue) corresponding to an organizer. The boundary cell type produces long-range signals that act to pattern the surrounding tissue according to concentration thresholds. Adapted from Meinhardt (1983). **(B)** A model for how the ad-ab boundary might influence leaf morphogenesis. A polarity field is invoked, Kper, that is perpendicular to the proximodistal axis (Kpd) and begins at the organ surface and ends at the ad-ab boundary. Growth is specifically reduced along Kper relative to the other two orthogonal directions Kpd and Kop. This assumption is sufficient for model simulations to generate a flattened leaf as well as other observed leaf morphologies, regardless of the initial shape of the primordium. Modified from Whitewoods et al. (2020).

Many leaf primordia likely start with an oval rather than circular-shaped cross-section simply because the auxin-response zone from which they form, i.e., the ad-ab boundary, is narrow and linear. In other words, even if auxin is concentrated at the ad-ab boundary in an irregular or circular distribution, because maximum auxin sensitivity lies along a narrow linear domain, the growth response will also be focused along this domain. According to this proposal an asymmetric primordium will arise whenever the domain of auxin accumulation is greater in diameter than the ad-ab boundary itself (Figure 4A). An example of an asymmetric shape at initiation is perhaps most obvious not only for sepal primordia in *Arabidopsis* flowers (Zhao et al., 2020) but is also apparent for *Arabidopsis* and tomato leaves (Zhao and Traas, 2021). Supporting this proposal, an inhibition of auxin transport in *pin1* mutants or by NPA treatment typically leads to wider but not necessarily thicker leaves (Okada et al., 1991), presumably due to a broader than usual auxin distribution, combined with a narrow linear response zone. Direct evidence that the initial configuration of the ad-ab boundary sets up organ growth patterns comes from experiments, in which ectopic KAN1 expression was induced in the *Arabidopsis* SAM (Caggiano et al., 2017) causing changes to the ad-ab boundary configuration (Figure 4B). Within the leaf primordia that subsequently developed, the boundary in organ founder cells could be correlated with an assortment of distinct morphologies indicating a fundamental role for the SAM ad-ab boundary in shaping leaf morphogenesis (Caggiano et al., 2017; Figure 4B). Does this mean that the ad-ab boundary plays no role in shaping growth after initiation and that mechanics might take over completely? An early model proposing a more ongoing and active role for the ad-ab boundary in shaping leaf development comes from early observations by Waites and Hudson of the *phantastica* (*phan*) mutant of Antirrhinum. In *phan* leaves, these authors found instances were abaxial cell types appeared ectopically on the adaxial side of the leaf. Associated with these ectopic cell types, ectopic leaf-like outgrowths appeared that were centered on the ectopic ad-ab boundary (Waites and Hudson, 1995). From these observations, Waites and Hudson proposed that the juxtaposition of adaxial and abaxial cell types might lead to the formation of an organizer that influences leaf growth non-cell autonomously through long-range signaling molecules (Waites and Hudson, 1995), analogously to the boundary-localized organizers of the fly wing (Figure 5A; Meinhardt, 1983; Diaz-Benjumea and Cohen, 1993). More recently, an extension of this idea has been applied to understanding the role of ad-ab boundaries in shaping the Carnivorous trap *Ultricularia gibba*. In this case, the young leaf primordia approximate a circle in cross-section and yet develop an elaborate morphology that can be predicted from the ad-ab boundary configuration (Whitewoods et al., 2020; therefore arguing against mechanical stress-feedback as the only regulating factor). The authors propose a hypothetical ortho-planar polarity field that extends between the epidermis and ad-ab boundary, meeting both at right angles (Figure 5B; Whitewoods et al., 2020). Simulations show that when growth rates are explicitly reduced along this axis compared to the other orthogonal axes, this is sufficient to generate various leaf morphologies, including those of carnivorous traps (Whitewoods et al., 2020). This is a striking result that matches the ortho-planar orientation of interphase microtubules and cellulose fibrils observed within growing leaves (which would be expected to reduce growth along this axis), as discussed above (Zhao et al., 2020). However, note that the close similarity in the proposed polarity field (Figure 5B) with the orientation of maximal mechanical tensions predicted by mechanical modeling (Figure 1C). Given that microtubules are already known to be mechano-sensitive, the question could be asked as to whether the hypothetical polarity field could correspond to mechanical stresses? While both models depend on primordium shape, the ortho-planar polarity field model also depends on the ad-ab boundary, i.e., ad-ab gene expression patterns. So even a symmetrical primordium will flatten over time according to the ortho-planar polarity field model, as long as an initial ad-ab boundary is present. This is not the case for the stress-feedback model (at least with no additional assumptions) since it entirely depends on organ shape. Since *WOX* genes promote leaf widening in part by promoting auxin synthesis (Zhang et al., 2020), one scenario is that post-initiation, the leaf margin serves as a source of auxin and combined with mechanical stress-feedback, this could account for an ongoing role for the ad-ab boundary in shaping leaf morphogenesis. Further evidence indicating such an ongoing role comes from examining developing leaves when differentiation is suppressed, as discussed below.

## Differentiation – the Gatekeeper of Morphogenetic Potential

**Figure 6:**
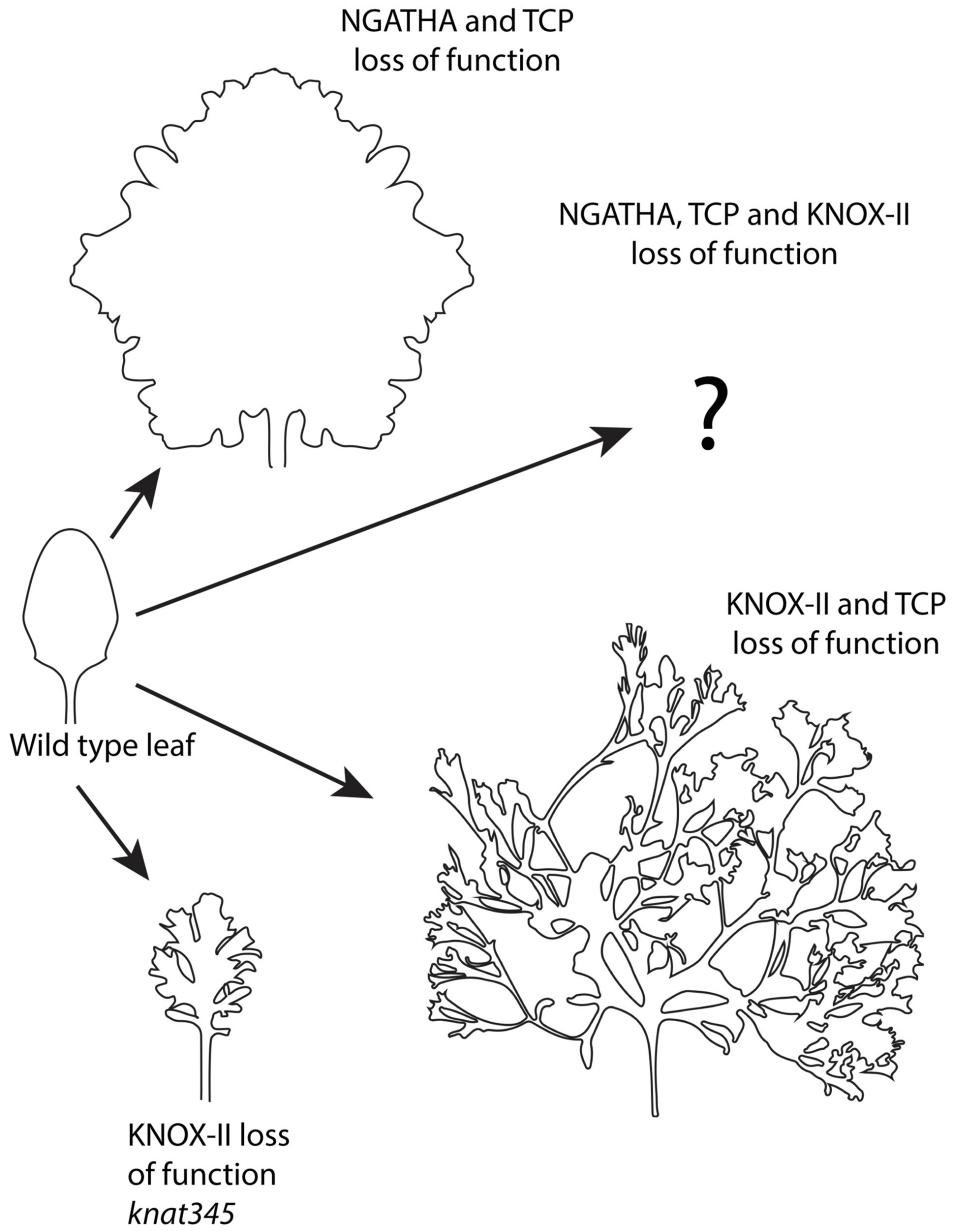
Continued organogenesis at the ad-ab boundary of the leaf, i.e. margin, depends on differentiation factors. Loss of function for different differentiation factors including NGATHA, TCP, and KNOX-II transcription factors in different combinations, results in differing degrees of continued growth and leaf dissection. Adapted from Furumizu et al. (2015), Alvarez et al. (2016), and Challa et al. (2021).

As previously discussed, the leaf margin represents a continuation of the ad-ab boundary or auxin responsive zone present in both the SAM and vascular system. Similar to the SAM PZ epidermis, the formation of new growth axes at the leaf margin is promoted by auxin, which is concentrated at serration sites by convergently polarized PIN1 much like at the SAM PZ. In the leaf, however, convergent patterns of PIN1 polarity require the function of CUC2, a NAC-domain transcription factor (Nikovics et al., 2006). *CUC2* expression is downregulated by auxin at PIN1 convergence sites but expressed in adjacent regions where it represses growth. Thus, auxin and CUC2 work in an opposite fashion to regulate contrasting growth patterns (Bilsborough et al., 2011). Why does not the *Arabidopsis* leaf margin produce fully fledged leaves like the SAM PZ? In other species, such as *Cardamine hirsuta*, leaves can be complex with secondary leaves developing from the margin of the primary leaves (Hay and Tsiantis, 2006). Studies have shown that the formation of leaflets critically depends on KNOX-I and KNOX-II class transcription factors, which act antagonistically to inhibit and promote the dissection of leaf margins into leaflets, respectively. For instance, KNOX-I expression, which is present in the SAM, is largely excluded from the simple leaves of *Arabidopsis* while, in *C. hirsuta,* the KNOX-I genes *chBP chSTM, ChKNAT2*, and *ChNAT6* are expressed in leaves, where they redundantly promote leaflet formation (Rast-Somssich et al., 2015). How do these genes influence leaf dissection? The KNOX-I genes suppress differentiation (Kierzkowski et al., 2019), i.e., the transition from a slow growing, slow dividing pluripotent state to a more specialized and determined state. Ectopically expressing the KNOX-I gene *STM* in *Arabidopsis* leaves for instance slows and prolongs the growth of distal tissues such that additional PIN1/CUC2-mediated serrations have the opportunity to arise. This pattern mimics the development of *C. hirsute* leaves except that *C. hirsuta* utilizes an additional transcription factor, RCO, which acts locally to enhance the suppression of growth at the sinuses such that they remain closer to the midrib (Kierzkowski et al., 2019). Expressing *RCO* under its own promoter in *Arabidopsis* combined with ectopic *STM* expression recapitulates *C. hirsuta* leaf morphology. As might be expected if *KNOX-II* genes promote differentiation, *Arabidopsis* loss of function mutants for the *KNOX-II* genes *KNAT3*, *KNAT4*, and *KNAT5* develop an enhanced serration or lobing phenotype that looks very similar to that caused by ectopic STM (Serikawa et al., 1997; Furumizu et al., 2015; Figure 6).

Other genes that regulate leaf differentiation encode members the GNATHA (NGA) and CINCINNATA class-TCP (CIN-TCP) transcription factor families. Strikingly, when the function of these proteins is jointly reduced leaf growth continues indefinitely (Alvarez et al., 2016; Figure 6). Importantly, this indeterminate growth appears driven from the margin since it depends on the activity of *WOX1* and *PRS*, which are expressed at the leaf margin. Knocking out NGATHA and CIN-TCP factors specifically at the leaf margin is also sufficient to promote indeterminate growth. Both cell divisions and auxin-dependent *MP* expression mark the marginal cells and cells marked with dye are found to be displaced away from the margin over time indicating continued production of leaf lamina tissue from the margin (Alvarez et al., 2016). Global gene expression profiles of older leaves suppressed for NGATHA and CIN-TCP function also indicate a differentiation state matching that of initiating wild type primordia. These observations, combined with the finding that only marginal *YUC1* expression can rescue the leaf width defect of *prs wox1* mutants (Zhang et al., 2020; mentioned earlier), supports a scenario in which lamina growth during the early stages of wild-type *Arabidopsis* leaf development is actively driven and shaped by cells at the margin, supporting the concept of a marginal meristem (Alvarez et al., 2016). How do the NGATHA and CIN-TCP transcription factors influence adaxial-abaxial patterning? As mentioned above, when adaxial or abaxial gene function is compromised, ectopic organs can form from adaxial or abaxial leaf tissues, respectively. However, the extent of these outgrowths is usually limited. If the differentiation program is suppressed at the same time as adaxial or abaxial gene activity is reduced, ectopic organogenesis is dramatically increased because a larger proportion of the leaf can respond and secondly, because the outgrowths themselves can grow indeterminately (Alvarez et al., 2016).

Finally, what happens to *Arabidopsis* leaves if knockdown of the CIN-TCP function is combined with knockdown of Class II KNOX function? In this case, super-compound leaves are formed as leaflets initiate indefinitely (Figure 6). This involves the activation of *KNOX-I* genes *KNAT2* and *KNAT6*, which positively regulates *CUC2* in a feedback loop (Challa et al., 2021). How an additional loss of NGATHA activity in this background might influence leaf morphology remains an interesting question to follow up.

## Conclusion and Future Perspective

Overall, the findings discussed immediately above reveal just how major a role the differentiation program plays in constraining plant morphogenetic potential and how its modulation can generate morphological diversity. Just as importantly though, the work discussed throughout this review highlights how several core patterning mechanisms integrate to generate that potential. Firstly, a mechanical stress feedback system not only physically supports the integrity of plant structures such as the leaves but also likely promotes their anisotropic growth (Figure 1). To form new growth axes therefore, stress reinforcement must be disrupted. This is accomplished by auxin, both through its ability to disrupt microtubule arrays and by its influence on cell wall enzymes. Such activity cannot operate unconstrained however. Auxin activity is restricted and patterned in several ways. The first corresponds to the action of adaxially and abaxially expressed transcription factors that limit auxin responsiveness to narrow boundary domains (Figure 2). This restriction profoundly influences lateral organ development by (1) restricting organogenesis to the shoot PZ (Figure 2), (2) shaping growth of the leaf primordium (Figure 4), and (3) promoting the propagation of ad-ab boundaries within new organs to potentiate iterative organogenesis (Figures 2, 6). Where the ad-ab boundary meets the epidermis, auxin is distributed periodically due to a feedback loop with its transport, which is somehow integrated with the microtubule-stress feedback system. This not only promotes a regular spacing between organ primordia, whether at the PZ or leaf margin, but also, an alignment between growth direction and PIN1 polarity. Finally, all this is kept in check *via* the action of genes that promote organ differentiation (Figure 6).

How broadly can the above narrative be applied? This is an interesting question for future studies. For instance, despite a lack of firm conclusions, early work on cellulose orientations in the cell walls of individual *Nitella* cells reveal extremely similar behavior to what is thought to occur in multicellular *Arabidopsis* meristem tissues, e.g., circumferential orientations in response to local loosening at the surface (mechanically induced laterals; Green, 1964; Green and King, 1966). In terms of ad-ab boundaries, similar configurations of adaxial and abaxial gene expression to that seen in *Arabidopsis* have now been observed in the shoot meristems of various fern species suggesting megaphyll leaves share a common developmental program and association with vascular cambium (Vasco et al., 2016; Zumajo-Cardona et al., 2019). While the downregulation of *KNOX-1* genes in leaf primordia is not observed in ferns, this has been interpreted as potentially reflecting delayed determinacy (Harrison et al., 2005), which seems supported by fern leaf morphology. While the data on organ initiation and PIN polarity convergences are sparse, examples are known from other eudicot and monocot species including tomato (Bayer et al., 2009), maize (Gallavotti et al., 2008) and *Brachypodium* (O’connor et al., 2014) and wounding experiments indicate the same type of inhibitory field model applied to *Arabidopsis* to explain organ spacing in abstract terms (Godin et al., 2020) is applicable to fern phyllotaxis (Steeves and Sussex, 1989). Given this, what steps were likely critical to leaf evolution? One precondition might be the presence of a circumferential ad-ab boundary around the SAM since this establishes position and ad-ab patterning, which is critical to developing a flattened organ oriented correctly with respect to the shoot axis (Caggiano et al., 2017). A second step may be the recruitment of factors such as the *YABBY* genes that promote a leaf-specific differentiation and growth program including the repression of SAM expressed genes (Sarojam et al., 2010).

Finally, while the narrative I have described attempts to paint a smooth picture, it is important to remember that the molecular mechanisms underlying many of the developmental processes described remain largely unknown. Of particular note, we do not understand how cell wall stress directions are apparently conveyed to microtubule orientations and the details for how this relates to PIN1 polarities. There are also many aspects of ad-ab boundary function that we do not understand. These and many other problems remain exciting challenges for the future.

## Author Contributions

The author confirms being the sole contributor of this work and has approved it for publication.

## Funding

The writing of this manuscript was supported by the Australian Government through the Australian Research Council’s Discovery Projects funding scheme (project DP180101149) awarded to MH.

## Conflict of Interest

The author declares that the research was conducted in the absence of any commercial or financial relationships that could be construed as a potential conflict of interest.

## Publisher’s Note

All claims expressed in this article are solely those of the authors and do not necessarily represent those of their affiliated organizations, or those of the publisher, the editors and the reviewers. Any product that may be evaluated in this article, or claim that may be made by its manufacturer, is not guaranteed or endorsed by the publisher.
